# Laboratory evaluation of transgenic *Populus davidiana*×*Populus bolleana* expressing *Cry1Ac* + *SCK*, *Cry1Ah3*, and *Cry9Aa3* genes against gypsy moth and fall webworm

**DOI:** 10.1371/journal.pone.0178754

**Published:** 2017-06-05

**Authors:** Liping Ding, Yajuan Chen, Xiaoli Wei, Mi Ni, Jiewei Zhang, Hongzhi Wang, Zhen Zhu, Jianhua Wei

**Affiliations:** 1 Beijing Key Laboratory of Agriculture Gene Resource and Biotechnology, Beijing Academy of Agriculture and Forestry Sciences, Beijing, P. R. China; 2 State Key Laboratory of Plant Genomics and National Center for Plant Gene Research (Beijing), Institute of Genetics and Developmental Biology, Chinese Academy of Sciences, Beijing, P. R. China; Institute of Plant Physiology and Ecology Shanghai Institutes for Biological Sciences, CHINA

## Abstract

Transgenic poplar lines ‘Shanxin’ (*Populus davidiana*×*Populus bolleana*) were generated via Agrobacterium-mediated transformation. The transgenic lines carried the expression cassettes of *Cry1Ac* + *SCK*, *Cry1Ah3*, and *Cry9Aa3*, respectively. The expression levels of the exogenous insect resistance genes in the transgenic lines were determined by Q-PCR and Western blot. Leaves of the transgenic lines were used for insect feeding bioassays on first instar larvae of the gypsy moth (*Lymantria dispar*) and fall webworm (*Hyphantria cunea*). At 5 d of feeding, the mean mortalities of larvae feeding on *Cry1Ac* + *SCK* and *Cry1Ah3* transgenic poplars leaves were 97% and 91%, while mortality on *Cry9Aa3* transgenic lines was about 49%. All gypsy moth and fall webworm larvae were killed in 7–9 days after feeding on leaves from *Cry1Ac* + *SCK* or *Cry1Ah3* transgenic poplars, while all the fall webworm larvae were killed in 11 days and about 80% of gypsy moth larvae were dead in 14 days after feeding on those from *Cry9Aa3* transgenic lines. It was concluded that the transgenic lines of *Cry1Ac* + *SCK* and *Cry1Ah3* were highly toxic to larvae of both insect species while lines with *Cry9Aa3* had lower toxicity,and *H*. *cunea* larvae are more sensitive to the insecticidal proteins compared to *L*. *dispar*. Transgenic poplar lines toxic to *L*. *dispar* and *H*. *cunea* could be used to provide Lepidoptera pest resistance to selected strains of poplar trees.

## Introduction

Poplars (*Populus* spp.) are economically important, rapidly growing trees. They are models in research on the genetic engineering of forest trees. Poplar is an important source of raw material for various wood-based products such as timber, pulp and fuel. Commercial plantations of poplars have expanded rapidly in recent years. In China, poplar plantations occupied 7.0 m ha in 2008 [1]. One of the major restrictive factors affecting poplar production is insect damage. Insects can severely reduce tree establishment and growth, and increase mortality. About 0.87 m ha of poplar were affected by defoliating insects and 0.71 m ha by stem borers in China during 2005 [2]. Pests of poplars are mainly lepidopterans and coleopterans [3].

Conventional breeding of *Populus* species has been very successful but it time consuming, labor intensive, and limited to innerspecies gene transmission. Modern genetic improvement methods, such as genetic modification can help overcome the limitations of conventional breeding techniques [4]. A variety of insect resistance genes, such as Bt (*Bacillus thuringiensis*) genes, proteinase inhibitor genes, and insect toxin genes, have been used in genetically modified poplar trees for pest control since McCown et al. (1991) first succeeded in stably transforming a Bt gene into poplar [5]. Stable expression of synthetic Bt toxin appears to be the most effective method for reducing damage and minimizing the tree mortality caused by insect pests. The proteins encoded by different Bt genes have specific toxicity to insect species in the orders Lepidoptera, Diptera, and Coleoptera. Two transgenic Lepidoptera-resistant poplar clones have been commercialized in China since 2002. One is *Populus nigra* containing *Cry1AC* and the other is transgenic hybrid poplar 741 (*P*. *alba* × (*P*. *davidiana* × *P*. *simonii* × *P*. *tomentosa*) containing both *Cry1AC* and the proteinase inhibitor gene (*API*) [6, 7]. The two commercialized transgenic poplars have shown significant efficacy in reducing the large-scale damage caused by some key target pests on poplar plantations [8].

The *Cry1Ac* gene is widely used for the control of Lepidoptera in poplar at present [9]. The experience has shown that the resistance conferred by a single Bt gene has the potential to break down as the target insect pest mutates and adapts to defeat the Bt trait [10]. To cope with the emergence of resistant insect population, there is great interest in finding novel insect-resistant gene with higher toxicity and/or wider spectrum [11]. In this study, we tried to develop new transgenic poplar with new insect genes other than *Cry1Ac*, in order to cope with the potential resistant insect in the future. There are over hundreds of new Bt genes which were cloned in recent years (see http://www.btnomenclature.info/). Taking into account the intellectual property rights of insect resistant genes, we had to use those genes that we obtain the permission to use, that are two new Bt genes (*Cry1Ah3* and *Cry9Aa3*) and one combination of *Cry1Ac* and *SCK*. We acquired three lines of transgenic ‘Shanxin’ poplar (*Populus davidiana*×*Populus bolleana*), produced via *Agrobacterium*-mediated transformation, which contained *Cry1Ac* + *SCK*, *Cry1Ah3* and *Cry9Aa3*, respectively. The leaves of transgenic lines with different insect-resistant genes were used for insect feeding trials performed on gypsy moth (*Lymantria dispar*) and fall webworm (*Hyphantria cunea*). The insecticidal effects of the foliage from the three transgenic poplars were compared.

## Materials and methods

### Plant material

The hybrid poplar ‘Shanxin’ (*Populus davidiana×Populus bolleana*) was used as the starting plant material. Prior to genetic transformation, microcuttings were micropropagated on a modified Murashige and Skoog medium, as described by Wang et al. (2011) [12].

### Vectors and *Agrobacterium* strains

**Fig 1:**
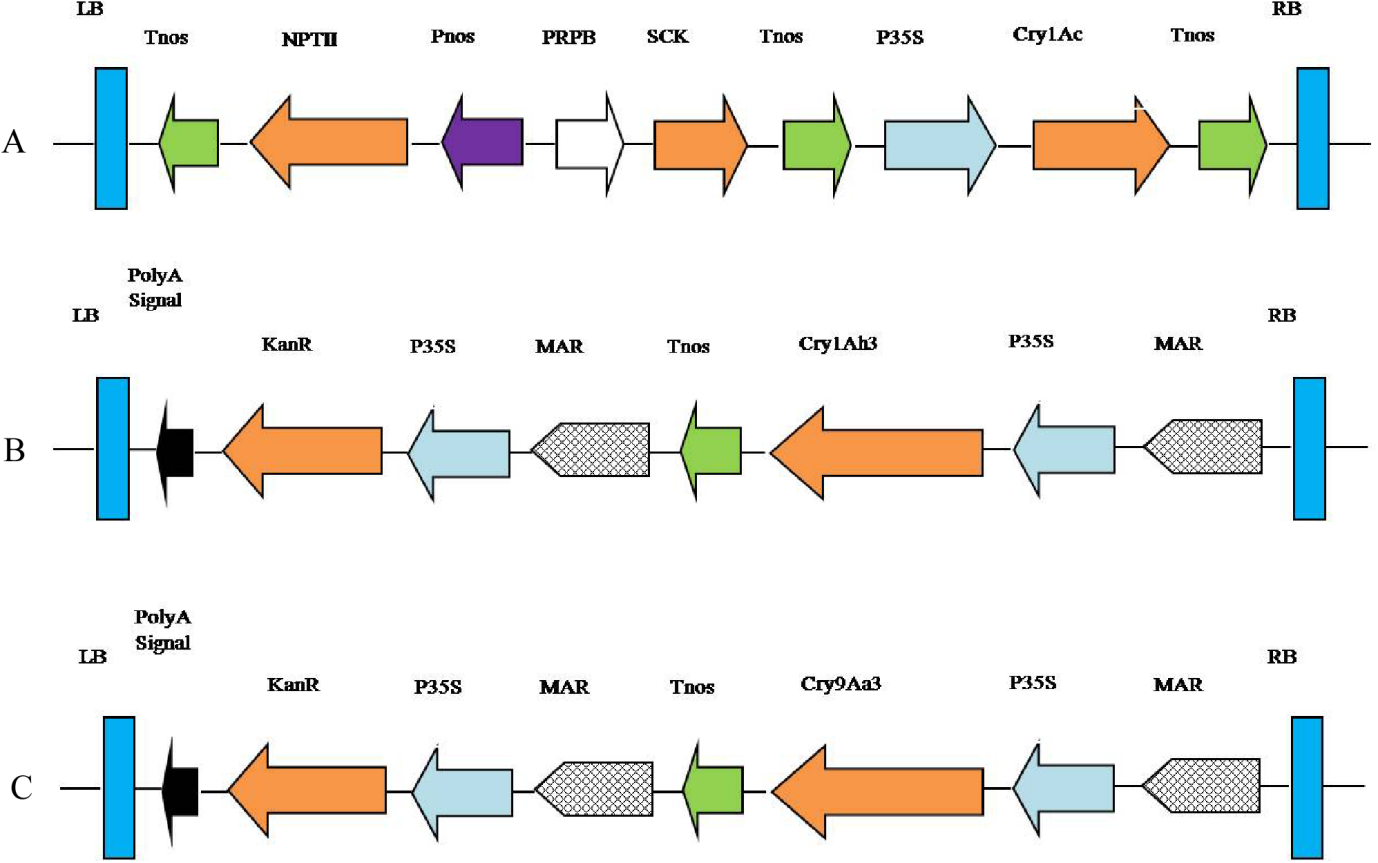
Frame diagrams of plant transformation vectors. **(A)** pCRPBSCK35SBt.(B) pP1Ah3. (C) pP9Aa3. LB: Left border; RB: right border; P35S: the cauliflower mosaic virus (CaMV) 35S promoter; NPT II or KanR: neomycin phosphotransferase II gene; Tnos or Poly A signal: poly-adenylation site of the nopalin synthase gene; MAR: matrix attachment region; Pnos: promoter of the nopalin synthase gene; PRPB: promoter of replication protein of CLCuV (Cotton leaf curl virus).

The binary vector pCRPBSCK35SBt, contains a *Cry1Ac* gene (1848 bp, see GenBank KF630361.1) driven by the cauliflower mosaic virus 35S promoter, and a *SCK* gene driven by RPB promoter. The binary vector pP1Ah3 contains a *Cry1Ah3* gene (2004 bp) driven by the cauliflower mosaic virus 35S promoter. The binary vector pP9Aa3 contains a *Cry9Aa3* gene (2043 bp) driven by the cauliflower mosaic virus 35S promoter. Each of the three vectors contains a neomycin phosphotransferase gene (*npt II*), as a selection marker gene. The vectors used in this paper were provided by the Zhen Zhu lab, at the Institute of Genetics and Development Biology, Chinese Academy of Sciences (CAS). The structures are illustrated in Fig 1.

### *Agrobacterium*-mediated transformation of poplar clones

The transformation of poplar clones was performed using the protocol reported by Wang et al. (2011) [12]. After regeneration and multiplication, the plantlets were acclimatized in a growth chamber before being transferred to a greenhouse.

### Plant genomic DNA isolation and PCR analysis

Genomic DNA was isolated from leaves (0.5 g) of transgenic and control plants using Plant DNA Isolation Reagent (TaKaRa). PCR analysis for the detection of the *Cry1Ac*, *SCK*, *Cry1Ah3* and *Cry9Aa3* was carried out using specific primer pairs as shown in Table 1. The PCR reactions were carried out in a total volume of 25 μl comprising 50 ng genomic DNA, 50 mM KCl, 10 mM Tris-HCl (pH 8.3), 1.5 mM MgCl_2_, 200 μM dNTPs, 1.25 units of Taq DNA polymerase, and 25 pmol of each primer. For PCR analysis, DNA was denatured at 94°C for 5 min followed by 30 cycles of amplification (94°C for 30 s. 56°C for 30 s, 72°C for 1 min) with final extension at 72°C for 10 min.

**Table 1:** Primers for PCR and Q-PCR.

Name	Primer (5’- 3’)	Fragment length	Annealing temperature
Cry1Ac-F	GCATTCCATACAACTGCTTG	1146bp	56°C
Cry1Ac-R	TTCCATAGGCGAACTCTGTT		
Cry1Ac-qPCR-F	CGCCTATGGAACCTCTTCTAAC	102bp	56°C
Cry1Ac-qPCR-R	GGTGGCACATTGTTGTTCTG		
SCK-F	GCACCATCTTCTTTGCTCTC	384bp	57°C
SCK-R	CATCTTCATCCCTGGACTTG		
SCK-qPCR-F:	AGCACCATCTTCTTTGCTCTC	100bp	54°C
SCK-qPCR-R	GTACCAGCACACACACCTTTA		
Cry1Ah3-F	TGGCTAAGAACAGCATCAAGC	1432bp	54°C
Cry1Ah3-R	GTGAATCCAGGAGAACATCGG		
Cry1Ah3-qPCR-F	ATCAGCACCTACACCGACTA	102bp	56°C
Cry1Ah3-qPCR-R	CCTGAACTGGTTGTACCTCAC		
Cry9Aa3-F	GGCTAAGTATCCTCTCGCTAACAA	1025bp	54°C
Cry9Aa3-R	GATGGTGAAACAGGCAAAGTCA		
Cry9Aa3-qPCR-F	CTATCCCAAATCCTAGGCCATC	108bp	56°C
Cry9Aa3-qPCR-R	CATACCAAACCCTAGCCCTATC		

### RT-qPCR assay

Three plants of each transgenic line and controls from 3-month-old seedlings in the greenhouse were selected randomly, and the fully expanded upper leaves of the seedling were collected, immediately put into liquid nitrogen, and transported to the laboratory. Total RNA was extracted from 100 mg of leaves using the RNeasy extraction kit (Qiagen, Germany). cDNA was synthesized using 5 μl of total RNA (500 ng) according to the Omniscript RT kit protocol (Qiagen) as described by the manufacturer and it was subsequently used as target for the qPCR reaction. According to the full sequence data of the targeted genes, fluorescence quantitative PCR primers were designed as shown in Table 1. With the previous reverse transcript cDNA as the template, 2 × Sybr Green qPCR Mix was used for fluorescence quantitative PCR. According to the cycle amounts that the fluorescent signal of each PCR reaction tube reaches in the designed threshold (CT value) and the standard curve developed with the use of standards that have a known expression amount, the abundance of the each Bt gene in the synthesized cDNA, with mRNA as transcription, was calculated from the standard curve [13].

### Western blot assay

Soluble proteins were extracted from fresh mature green leaves of 3-month-old transgenics and wild type plants in 200 mM Tris-HCl pH8.0 with 0.2% insoluble polyvinylpyrrolidone at 4°C. Approximately 40 ug of total protein from individual lines was separated on SDS-PAGE and blotted. Electrotransfer on a polyvinylidene difluoride (PVDF) membrane (Immobilon, Millipore) was performed using the mini protean-II apparatus (Bio Rad, USA). A purified anti-beta actin antibody (ab129348) was used as standard. Polyclonal rabbit anti-Cry1Ac antiserum (ABCAM, ab51586) was used as the primary antibodies for Cry1Ac expression, monoclonal rabbit anti-Cry1Ah3 antiserum (provided by the Institute of Genetics and Development Biology, CAS) was used for Cry1Ah3, and polyclonal rabbit anti-Cry9Aa3 antiserum (provided by the Institute of Genetics and Development Biology, CAS) was used for Cry9Aa3. Detection was performed with a chemiluminescent western blotting kit (Roche, USA), using secondary antibodies conjugated to a phosphatase. Due to lack of availability anti-SCK antibody, Western blot assay of SCK was not conducted.

### Insect bioassay testing

We conducted insect bioassays to observe the responses of *L*. *dispar* and *H*. *cunea* larvae to leaves of transgenic and control poplars. The first instar larvae of *L*. *dispar* and *H*. *cunea* were provided by Research Institute of Forest Ecology, Environmental and Protection, Chinese Academy of Forestry (CAF). The spawns were collected in the nursery in Chinese Academy of Forestry. The newly hatched instar larvae of *L*. *dispar* and *H*. *cunea* were directly provided for us to conduct the feeding experiment. The fresh leaves of the about 3-month-old seedlings of the transgenic lines and controls were collected from the greenhouse. They were handpicked, washed with distilled water, and kept on moist filter paper in Petri dishes. A total of 30 first instar larvae were transferred to each Petri dish. The plates were held at 25±2°C under a 14-h light 10-hr dark photoperiod. The fresh leaves were added into each plate every 2 days. The insect mortality was checked at several times after feeding. The test for each transgenic line was repeated three times.

### Statistical analysis

Excel was used to store and process the experimental data. SPSS version 13.0 was used for multiple comparisons.

## Results

### PCR detection of transgenic plants

**Fig 2:**
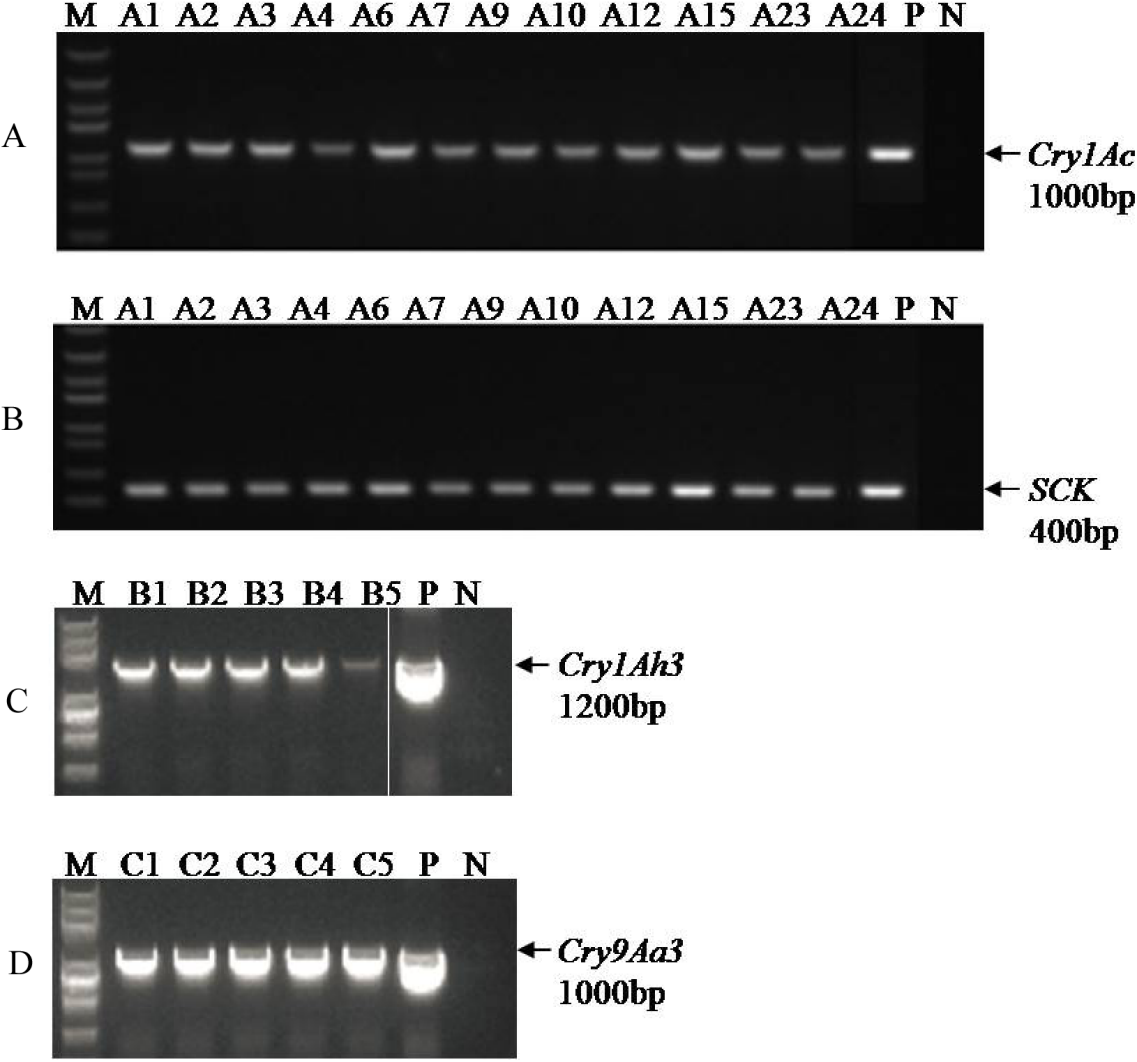
PCR detection of transgenic poplar. (A) and (B) PCR analysis for the presence of the *Cry1Ac* and *SCK* gene in transgenic plants. (C) PCR analysis for the presence of the *Cry1Ah3* gene in transgenic plants. (D) PCR analysis for the presence of the *Cry9Aa3* gene in transgenic plants. M: DNA fragments size marker (Trans5K); P: plasmid (positive control); N: wild type plants (negative control). The arrows indicate the expected PCR products.

The *Agrobacterium*-mediated transformation method produced some kanamycin resistant lines from independent transformation events of all the three plasmids. The specific primers were adopted to PCR amplification of the target gene in each of transgenic lines. Plasmid was used as positive controls, while DNA extracted from untransformed plants was used as a negative control. The PCR amplification results (Fig 2) showed that 12 lines of plasmid pCRPBSCK35SBt were obtained and named as the A series; 5 transgenic lines of pP1Ah3 were obtained and named the B series (B1 to B5); 5 transgenic lines of plasmid pP9Aa3 were obtained and named the C series (C1 to C5). The PCR assay verified that the target genes were integrated into the poplar genome.

### Qualitative PCR detections of transgenic lines

The expression of *Cry1Ac*, *Cry1Ah3* and *Cry9Aa3* were verified at the RNA level by RT-qPCR. RNA samples were isolated from the green leaves of transgenic lines. No amplification signal was observed in the controls (untransformed plants). According to the detected CT value, the computed starting transcription abundance of the Bt gene in the samples (every 100 mg of fresh leaves) is listed in Table 2. It shows that the transcription abundance of *Cry1Ac* ranged from 6.02×10^4^ in line A15 to 8.27×10^6^ in A4; abundance of *Cry1Ah3* ranged from 5.95×10^5^ in line B5 to 3.66×10^6^ in B3; and abundance of *Cry9Aa3* ranged from 7.17×10^5^ in C4 to 2.18×10^6^ in C1. Statistical analysis shows that significant differences existed in the transcription abundances of the Bt genes among the different transgenic lines with the same vector.

**Table 2:** Transcript abundance of the *Cry1Ac*, *SCK*, *Cry1Ah3* and *Cry9Aa3* genes detected by qRT-PCR.

Line	Transcript abundance of the Cry1Ac gene	Transcript abundance of the SCK gene	Line	Transcript abundance of the Cry1Ah3 gene	Line	Transcript abundance of the Cry9Aa3 gene
CK	0.00	0.00	CK	0.00a	CK	0.00a
A1	7.94×10^5^	4.65×10^5^	B1	1.73×10^6^	C1	2.18×10^6^
A2	1.80×10^6^	1.22×10^5^	B2	2.38×10^6^	C2	1.34×10^6^
A3	2.40×10^6^	1.91×10^5^	B3	3.66×10^6^	C3	1.66×10^6^
A4	8.27×10^6^	3.41×10^4^	B4	7.67×10^5^	C4	7.17×10^5^
A6	1.85×10^6^	6.99×10^4^	B5	5.95×10^5^	C5	7.24×10^5^
A7	1.73×10^6^	4.01×10^4^				
A9	8.91×10^5^	1.45×10^4^				
A10	6.99×10^5^	4.04×10^4^				
A12	1.70×10^6^	3.29×10^4^				
A15	6.02×10^4^	6.02×10^4^				
A23	5.77×10^5^	1.17×10^4^				
A24	3.58×10^6^	7.67×10^4^				

CK means control, which is wild type of poplar.

### Western blot assay of Bt proteins in the transgenic lines

**Fig 3:**
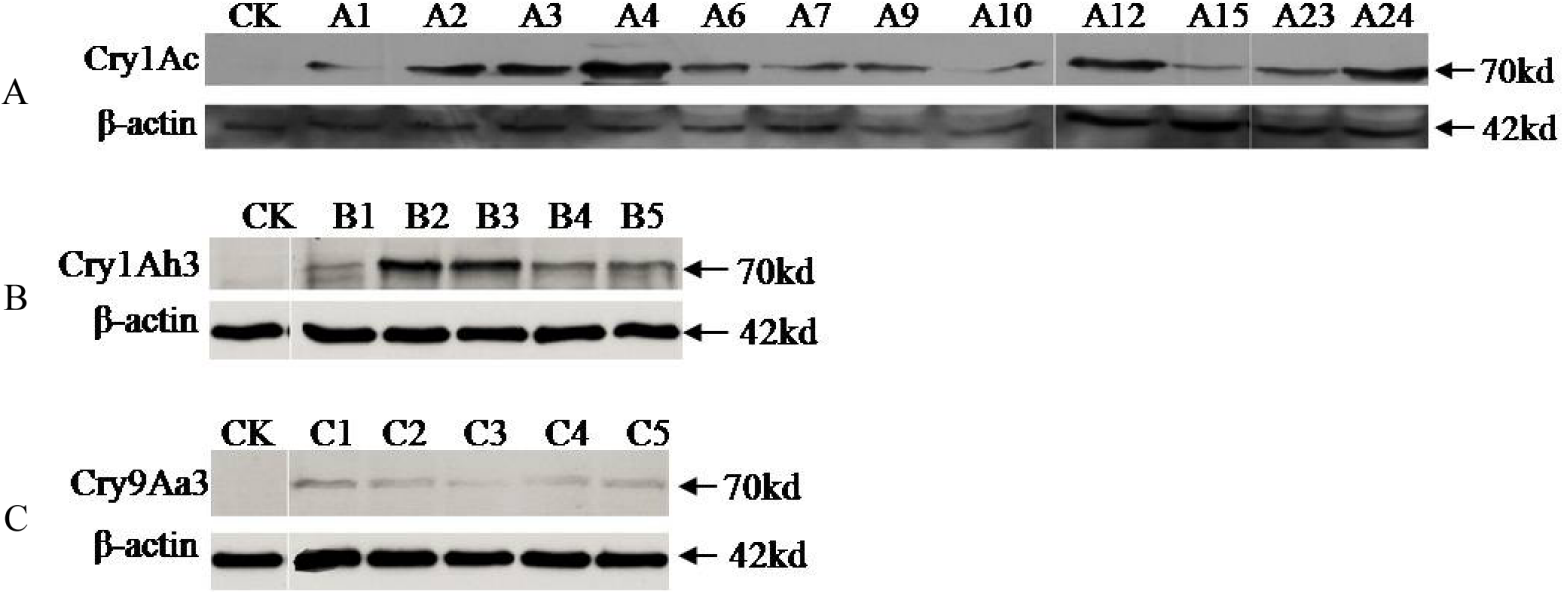
Western blot assay of Bt proteins in the transgenic lines. (A) Western blot assay for the expression of Cry1Ac. (B) Western blot assays for the expression of Cry1Ah3. (C) Western blot assays for the expression of Cry9Aa3,. The arrows indicate the expected protein products (70kD). CK means control, which is wild type of poplar.

A total of 12 transgenic lines containing *Cry1Ac* + *SCK*, 5 lines containing *Cry1Ah3*, and 5 lines containing *Cry9Aa3* were analyzed by Western blot assay to examine the protein expression level of Cry1Ac, Cry1Ah3 and Cry9Aa3 in the different lines. One well-defined band was detected at about 70 kDa in all test lines, which was consistent with the predicted molecular weight values from the DNA sequences of the corresponding *Cry1Ac*, *Cry1Ah3* and *Cry9Aa3* genes, respectively. Different transgenic lines had variable levels of target protein expression (Fig 3). Within the same transgenic line series, the target Bt protein expression levels is approximately consistent with the transcription abundance detected by qualitative PCR in exact lines. For example, in line series A, the line A4 had the strongest signal in the Western blot assay (Fig 3A) and the highest transcription abundance in the Q-PCR assay (Table 2). Line A15 had a relatively weaker signal in the Western blot assay (Fig 3A) and low transcription abundance (Table 2). The signals of Cry9Aa3 were all much weaker than those of Cry1Ac or Cry1Ah3, which may have resulted from the weak sensitivity of anti-Cry9Aa3 antibody.

### Insect feeding experiment of transgenic lines

Three transgenic lines transformed with different protein, which showed relatively high expression levels of target genes, were selected for the insect feeding experiment. A total of 30 first instar larvae of *L*. *dispar* or *H*. *cunea* were fed on the leaves of twelve independently derived transgenic lines (A3, A4, A24, B1, B2, B3, C1, C2 and C3) with leaves from non-transgenic poplar plants as controls.

**Fig 4:**
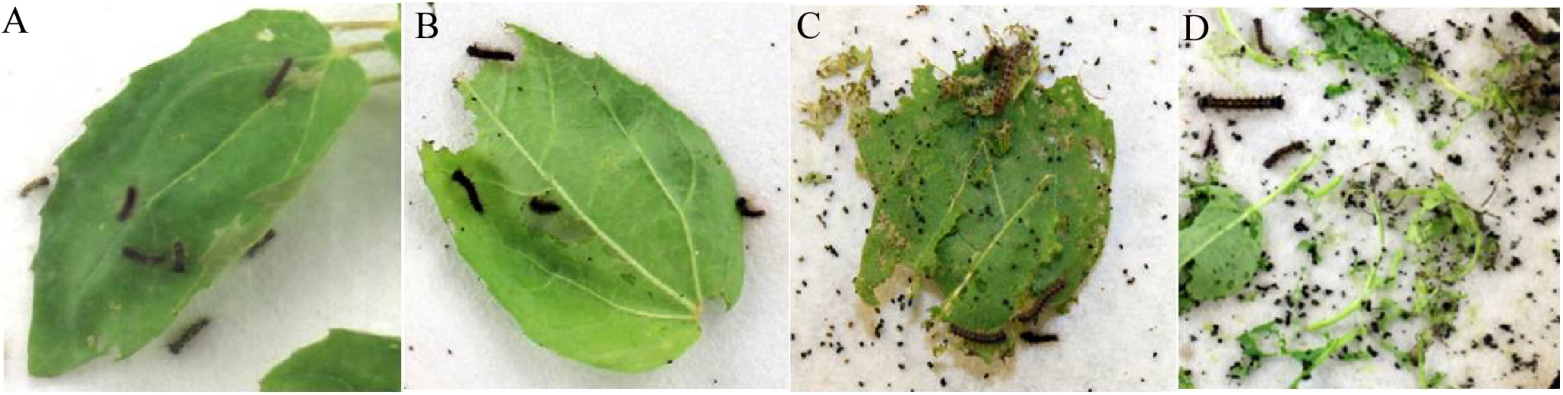
Poplar leaf bioassay against gypsy moth larvae after 7 d of feeding. (A) A3 (Cry1Ac and SCK). (B) B1 (Cry1Ah3). (C) C1 (Cry9Aa3). (D) wild type as control.

In the *L*. *dispar* feeding experiment, mortality after 3 d, 4 d, 5 d, 7 d, 9 d, 11 d, 13 d and 15 d of feeding is shown in Table 3. There was a significant difference in the larval mortalities among the three transgenic lines and the control. At 5 d of feeding the mean mortalities of larvae feeding on series A and B leaves were 97% and 91%, respectively, while mortality on series C was about 49%. At 7 d of feeding, all the larvae feeding on the leaves of series A and B were dead except those on B2 leaves (the mortality is about 95%), while 54% of larvae on leaves of series C were dead. The effects of different insecticidal proteins on gypsy moth larvae were showed in Fig 4 at 7 d. The mean mortalities on series A at 5 d and 7 d were somewhat higher than those on series B, the difference was not significant. Series A and B, representing *Cry1Ac* + *SCK* and *Cry1Ah3*, have similar high toxicity against *L*. *dispar* larvae. There was 80% mortality of *L*. *dispar* larvae on the series C leaves at 15 d while the control mortality was 45%. Series C (*Cry9Aa3*) lines were relatively less toxic to *L*. *dispar* larvae.

**Table 3:** Mortalities (%) of gypsy moth larvae fed with leaves of three different transgenic lines.

Lines	Total mortality (%) on days after feeding															
	3rd day		4th day		5th day		7th day		9th day		11th day		13th day		15th day	
	Line mean	Treatment mean	Line mean	Treatment mean	Line mean	Treatment mean	Line mean	Treatment mean	Line mean	Treatment mean	Line mean	Treatment mean	Line mean	Treatment mean	Line mean	Treatment mean
CK	0.0±0.0a	0.0±0.0	5.6±1.9a	5.6±1.9	6.7±0.0a	6.7±0.0	7.8±1.9a	7.8±1.9	14.4±1.9a	14.4±1.9	26.7±5.1a	26.7±5.1	38.9±6.7a	38.9±6.7	44.5±6.9a	44.5±6.9
A3	24.4±5.1d	23.7±4.7	55.6±6.9de	58.2±4.6	97.8±1.9e	97.4±1.3	100.0±0.0e	100.0±0.0	100.0±0.0d	100.0±0.0	100.0±0.0d	100.0±0.0	100.0±0.0c	100.0±0.0	100.0±0.0c	100.0±0.0
A4	25.6±3.9d		57.8±5.1def		94.4±1.9e		100.0±0.0e		100.0±0.0d		100.0±0.0d		100.0±0.0c		100.0±0.0c	
A24	21.1±5.1d		61.1±1.9ef		100.0±0.0e		100.0±0.0e		100.0±0.0d		100.0±0.0d		100.0±0.0c		100.0±0.0c	
B1	3.3±0.0ab	7.0±1.7	67.8±5.8f	64.8±6.2	96.7±3.3e	91.1±5.1	100.0±0.0e	96.3±2.3	100.0±0.0d	98.1±1.7	100.0±0.0d	100.0±0.0	100.0±0.0c	100.0±0.0	100.0±0.0c	100.0±0.0
B2	4.4±1.9ab		57.8±5.1def		80.0±8.8d		88.9±6.9d		94.4±5.1d		100.0±0.0d		100.0±0.0c		100.0±0.0c	
B3	13.3±3.3c		68.9±7.7f		96.7±3.3e		100.0±0.0e		100.0±0.0d		100.0±0.0d		100.0±0.0c		100.0±0.0c	
C1	8.9±1.9bc	3.7±0.6	47.8±1.9cd	41.1±3.0	53.3±0.0c	48.9±3.0	64.4±9.2c	54.4±7.0	71.1±3.9c	60.8±7.0	72.2±1.9c	66.7±2.4	77.8±1.9b	74.5±3.6	81.1±1.9b	80.0±4.1
C2	2.2±0.0a		36.7±3.3b		43.3±3.3b		46.7±6.7b		55.6±10.1b		67.8±1.9c		76.7±5.1b		80.0±1.9b	
C3	0.0±0.0a		38.9±3.9bc		50.0±5.8bc		52.2±5.1b		55.6±6.9b		60.0±3.3b		68.9±3.9b		78.9±8.4b	

The statistical analysis was conducted following the ANOVA. Fisher’s least square difference (LSD) test was used to compare the means. Within each column, means with the same lowercase letter are not significantly different (*P* = 0.05). CK means control, which is wild type of poplar.

In the *H*. *cunea* larvae feeding experiment, mortality at 3 d, 4 d, 5 d, 7 d, 9 d and 11 d of the feeding trial is shown in Table 4. Similar to the *L*. *dispar* results, the transgenic lines with *Cry1Ac* + *SCK* and *Cry1Ah3* were highly toxic to *H*. *cunea*, while the *Cry9Aa3* lines showed relatively lower toxicity.

**Table 4:** Mortalities (%) of fall webworm larvae fed with leaves of three different transgenic lines.

Lines	Total mortality (%) on days after feeding											
	3rd day		4th day		5th day		7th day		9th day		11th day	
	Line mean	Treatment mean	Line mean	Treatment mean	Line mean	Treatment mean	Line mean	Treatment mean	Line mean	Treatment mean	Line mean	Treatment mean
CK	0.0±0.0a	0.0±0.0	14.4±3.9a	14.4±3.9	20.0±0.0a	20.0±0.0	20.0±0.0a	20.0±0.0	22.2±3.9a	22.2±3.9	36.7±3.3a	36.7±3.3
A3	43.3±3.3efg	47.8±3.4	91.1±5.8e	91.1±7.0	100.0±0.0d	100.0±0.0	100.0±0.0d	100.0±0.0	100.0±0.0b	100.0±0.0	100.0±0.0b	100.0±0.0
A4	53.3±5.1g		93.3±8.4e		100.0±0.0d		100.0±0.0d		100.0±0.0b		100.0±0.0b	
A24	46.7±1.9fg		88.9±6.9de		100.0±0.0d		100.0±0.0d		100.0±0.0b		100.0±0.0b	
B1	34.4±7.7cde	35.9±5.4	82.2±6.9de	71.9±7.0	96.7±5.8d	97.0±3.6	100.0±0.0d	100.0±0.0	100.0±0.0b	100.0±0.0	100.0±0.0b	100.0±0.0
B2	35.6±5.1def		76.7±8.4d		100.0±0.0d		100.0±0.0d		100.0±0.0b		100.0±0.0b	
B3	37.8±3.3def		56.7±5.8c		94.4±5.1d		100.0±0.0d		100.0±0.0b		100.0±0.0b	
C1	26.7±3.8bcd	21.9±2.5	36.7±1.9b	27.8±3.2	56.7±3.3c	45.2±4.5	92.2±8.4d	82.9±6.3	96.7±5.8b	96.7±5.3	100.0±0.0b	100.0±0.0
C2	15.6±1.9b		23.3±5.8a		35.6±1.9b		72.2±3.9b		100.0±0.0b		100.0±0.0b	
C3	23.3±1.9bc		23.3±1.9a		43.3±8.4b		84.4±6.7c		93.3±10.2b		100.0±0.0b	

The statistical analysis was conducted following the ANOVA, Fisher’s least square difference (LSD) test was used to compare the means. Within each column, means with the same lowercase letter are not significantly different (*P* = 0.05). CK means control, which is wild type of poplar.

Comparison of Table 3 and Table 4 data shows that *H*. *cunea* larvae are more sensitive to all of the insecticidal proteins compared to *L*. *dispar*. Mortalities of *H*. *cunea* larvae reached 100% sooner than those of *L*. *dispar* on both series A and B. About 20% of the *L*. *dispar* larvae survived 15 d on series C, while all *H*. *cunea* larvae on series C were dead at 11 d.

## Discussion

In order to provide new insect resistant genes forLepidoptera control, the toxicity of two new Bt proteins, *Cry9Aa3* and *Cry1Ah3*, and combination of *Cry1Ac* and *SCK* to Lepidoptera was determined in transgenic poplars. Both *Cry9Aa3* and *Cry1Ah3* were cloned in 2010 (see http://www.btnomenclature.info/). It was reported that Cry9Aa3 had high insecticidal activity against *Plutella xylostella* and *Ostrinia furnacalis* [14]. There are no published reports on the insecticidal activities of the Cry1Ah3 protein. *CpTI* (Cowpea Trypsin Inhibitor) is one alternative to Bt genes and was reported to be toxic to the forest tent caterpillar (*Malacosoma disstria*), gypsy moth (*L*. *dispar*) and willow moth (*Stilpnotia candida*) [15]. *SCK* is derived from *CpTI*, which was fused with the signal peptide sequences and endoplasmic reticulum location sequences at 5' end and 3' end of *CpTI*[16]. *SCK* combined with *Cry1Ac* conferred high insect resistance in transgenic rice and cotton[16]. There are no reports on application of *Cry9Aa3*, *Cry1Ah3* and *Cry1Ac* +*SCK* to provide insect resistance to transgenic poplar. To cope with possible emergence of resistant Lepidoptera resulted from application of single *Cry1Ac* gene, new insect-resistant genes or stacking of different genes are considered to be one of solutions. We compared the toxicity of *Cry9Aa3*, *Cry1Ah3* and *Cry1Ac* + *SCK* in transgenic poplar against *L*. *dispar* and *H*. *cunea* using feeding assays. The results showed that transgenic poplars with *Cry1Ac* + *SCK* or *Cry1Ah3* have similar high resistance to larvae, while those with *Cry9Aa3* have weaker resistance. All *L*. *dispar* and *H*.*cunea* larvae were killed in 7–9 d after feeding on leaves from *Cry1Ac* + *SCK* or *Cry1Ah3* transgenic poplars. Our results indicate that *Cry1Ah3* and *Cry1Ac* + *SCK* could be used as new insect-resistant genes to provide poplar with high resistance against major lepidopteran pests.

*H*. *cunea* larvae were more sensitive, in this study, to insect-resistant proteins than larvae of *L*. *dispar*. Similar results were reported by Gao et al. (2004), who found *H*. *cunea* larvae were more sensitive than *L*. *dispar* to transgenic poplar ‘741’ carrying *Cry1Ac* and *API* (arrowhead proteinase inhibitor) [17].

*L*. *dispar* is a key lepidopteran pest damaging forests in many countries [18]. *H*. *cunea* is a worldwide quarantine pest. These two species cause serious economic losses and environmental harm. Poplar ‘Shanxin’ (*Populus davidiana*×*Populus bolleana*) is a major plantation species of poplar in Northeast China. The transgenic poplars ‘Shanxin’ with high resistance against both *L*. *dispar* and *H*. *cunea* could be used in future for protection against Lepidoptera feeding damage.
